# Exploring the expression and clinical significance of the miR-140-3p-HOXA9 axis in colorectal cancer

**DOI:** 10.1007/s00432-023-05592-3

**Published:** 2024-01-29

**Authors:** Wei Cui, Xueliang Bai, Zhongyuan Bai, Fengxin Chen, Jing Xu, Wenqi Bai, Yanfeng Xi

**Affiliations:** 1 Department of Pathology, Shanxi Province Cancer Hospital/Shanxi Hospital Affiliated to Cancer Hospital, Chinese Academy of Medical Sciences/Cancer Hospital Affiliated to Shanxi Medical University, Taiyuan, 030013 Shanxi People’s Republic of China; 2 School of Basic Medicine, Shanxi Medical University, Taiyuan, 030001 Shanxi People’s Republic of China; 3 First Clinical Medical School, Shanxi Medical University, Taiyuan, 030001 Shanxi People’s Republic of China; 4 Department of Colorectal Surgery, Shanxi Province Cancer Hospital/Shanxi Hospital Affiliated to Cancer Hospital, Chinese Academy of Medical Sciences/Cancer Hospital Affiliated to Shanxi Medical University, Taiyuan, 030013 Shanxi People’s Republic of China

**Keywords:** Colorectal cancer, miR-140-3p, HOXA9, Prognostic biomarker

## Abstract

**Purpose:**

This study aims to investigate the expression patterns and clinical significance of miR-140-3p and homeobox A9 (HOXA9) in colorectal cancer (CRC) selected by bioinformatic study, while elucidating their potential interplay.

**Methods:**

The microRNA expression profiles of paired colorectal cancer and matched normal tissues were retrieved from the Gene Expression Omnibus Database. Differentially expressed microRNAs and microRNA candidates were filtered and subjected to further analysis. Clinicopathological data, along with paraffin-embedded samples of colorectal tumor tissues were collected to facilitate comprehensive analysis. Expression levels of miR-140-3p and HOXA9 were quantified using qRT-PCR and immunohistochemistry. Survival rates were determined using the Kaplan–Meier method, and the COX regression model was utilized to identify independent prognostic factors that impact the overall prognosis.

**Results:**

MiR-140-3p was significantly downregulated in colorectal tumors compared to normal tissue, and HOXA9 was identified as a previously unreported potential downstream target. HOXA9 expression was elevated in tumors compared to normal tissues. Reduced miR-140-3p expression was associated with lymph node metastasis, while high HOXA9 expression correlated with both lymph node metastasis and lympho-vascular invasion. Patients with low miR-140-3p and high HOXA9 expression had a poorer prognosis. HOXA9 was identified as an independent risk factor for CRC patient survival.

**Conclusion:**

The miR-140-3p-HOXA9 signaling disruption is closely linked to lymph node metastasis and unfavorable prognosis in CRC. This axis shows promise as a clinical biomarker for predicting the CRC patient survival and a potential therapeutic target.

**Supplementary Information:**

The online version contains supplementary material available at 10.1007/s00432-023-05592-3.

## Introduction

Colorectal cancer (CRC) is the third most prevalent cancer globally with substantial morbidity and mortality (Sung et al. 2021). Surgical intervention has proven effective in managing early-stage CRC patients, while most patients were diagnosed at advanced stage or with distant metastases (Paczek et al. 2022). Despite achievement in diagnosis and therapeutic approaches for colorectal cancer over the past decade, the 5-year survival rate for advanced patients remains unsatisfactory. Consequently, it is urgent to delve deeper into the underlying pathogenesis of CRC and develop novel therapeutic strategies to address the unmet healthcare challenge.

microRNAs (miRNAs) are short, non-coding RNA molecules consisting of approximately 18–24 nucleotides. They play a significant role in gene expression regulation by binding to the 3′ untranslated regions (3′-UTRs) of target mRNA molecules, leading to mRNA degradation or inhibition of translation. Subsequently, miRNAs exert a negative regulatory effect on target genes. miRNAs have been implicated as crucial regulators of tumor suppressor genes and oncogenes (Chen et al. 2019b; Ji et al. 2014), and are involved in various biological processes, including cell proliferation, apoptosis, and cell cycle regulation (Chen et al. 2019a).

Extensive research has underscored the strong correlation between dysregulated miRNAs and the progression of colorectal cancer. These aberrantly expressed miRNAs hold promise as novel targets for CRC therapies (Ciesla et al. 2011). Notably, miR-140-3p has been observed to display altered expression patterns in various cancer types, including lung cancer, breast cancer, spinal cord chordoma, and hepatocellular carcinoma and so on (Taheri et al. 2023). miR-140-3p functions as a tumor suppressor, playing a crucial role in various cellular processes and influencing signaling pathways. Its impact on tumor development involves regulating downstream target genes. For instance, it targets ATP8A1 in non-small cell lung cancer and tripartite motif 28 in breast cancer (Dong et al. 2016; Zhou et al. 2019). Additionally, studies indicate that miR-140-3p can influence drug resistance by targeting the pregnenolone X receptor in hepatocellular carcinoma cells (Li et al. 2018). In colorectal cancer (CRC), lower plasma exosome miR-140-3p levels are observed in patients compared to healthy individuals. This downregulation is also seen in liver metastasis (Liu et al. 2021). In cell lines, miR-140-3p has been shown to inhibit cell proliferation, migration, and invasion by targeting BCL9 and promoting apoptosis by targeting BCL2 and PD-L1 (Jiang et al. 2019; Liu et al. 2021). Nonetheless, the specific expression profile, clinical implications and signaling networks of miR-140-3p in the context of CRC remain largely unexplored.

The homeobox gene (HOXA) family comprises a group of transcriptional regulators with highly conserved sequences. The homeobox A9 (HOXA9), a member of the HOXA family, has been extensively investigated, mainly in acute myeloid leukemia (AML). HOXA9 plays a critical role in promoting blood cell expansion, altering cellular differentiation, and driving malignancies (Li et al. 2013; Talarmain et al. 2022). In recent years, there has been a growing interest in the role of HOXA9 in solid tumors, and its aberrant expression is closely associated with patient prognosis (Tang et al. 2022). Notably, a study by Watanabe et al. revealed that HOXA9 overexpression in colorectal cancer tissues was linked to lymph node metastasis (Watanabe et al. 2018).

In this study, we have identified a significantly downregulated miRNA, miR-140-3p, in colorectal cancer, and have discovered HOXA9 as a novel target of miR-140-3p. Furthermore, this research provides clinical data supporting the significance of miR-140-3p and HOXA9 expression and their potential applications in the context of CRC.

## Materials and methods

### Bioinformatics analysis

miRNA expression datasets (GSE122187, GSE110402, GSE39845) were retrieved from the GEO database (https://www.ncbi.nlm.nih.gov/geo/). The raw data were analyzed by R. The ratio of miRNA expression in colorectal tumors compared to adjacent normal tissues was calculated as the fold change (Fc), and differentially expressed miRNAs were defined as those with |LogFc|> 1 and *p* < 0.01. To identify commonly differentially expressed miRNAs among the three datasets, we employed the VENN drawer online tool. To analyze the expression of miR-140-3p and its potential target genes, we utilized the ENCORI database (https://starbase.sysu.edu.cn/), a comprehensive online resource for miRNA analysis. Additionally, the GEPIA2 database (gepia2.cancer-pku.cn) and the TIMER database (https://cistrome.shinyapps.io/timer/) were utilized to investigate the expression of HOXA9 in colorectal cancer and normal tissues, as well as to explore the correlation between miR-140-3p and HOXA9. Finally, we employed the ENCORI database to analyze the prognostic significance of miRNAs and potential regulatory genes.

### Clinical data collection

We collected paraffin-embedded tissues and adjacent normal paraffin tissues from patients who underwent surgical resection for pathologically confirmed colorectal adenocarcinoma at Shanxi Cancer Hospital between January 2019 and June 2019. The patient cohort consisted of 109 males and 101 females, with ages ranging from 28 to 89 years and a median age of 62 years. Among these cases, 193 had complete follow-up information. Our inclusion criteria were as follows: (1) no preoperative antitumor treatment administered through any route; (2) complete clinical records available; (3) confirmation of adenocarcinoma through pathological examination of colorectal cancer biopsies and absence of tumor cell infiltration in the adjacent para-cancerous tissue.

### Tissue microarray preparation

A tissue microarray block instrument with a 2 mm aperture was used to create the paraffin microarray chip. A total of 210 colorectal cancer samples were re-evaluated based on hematoxylin–eosin stained (HE) sections, and the typical tumor areas were identified and marked on the pathological sections under a microscope. Using a puncture needle, the corresponding tissues marked on the HE sections were carefully picked and punched into the prepared blank tissue chip. Once the chips were prepared, they were placed upside down on the surface of a blank slide. Subsequently, the chips were heated at 55 °C in a drying oven for 7 min, followed by cooling down at room temperature outside the oven for 7 min. This heating and cooling process was repeated until the picked tissue was fully in contact with the paraffin on the chip.

### Immunohistochemical staining

All tissue specimens were fixed in 10% neutral formalin, dehydrated in a gradient of alcohol, and then made transparent in xylene. Subsequently, the specimens were embedded in paraffin. The paraffin tissue microarrays used for immunohistochemical (IHC) staining had a thickness of 3 μm. For the automatic immunohistochemical staining, the Roche VENTANA BenchMark XT Immunohistochemical Stainer was employed. The rabbit anti-human HOXA9 monoclonal antibody (Catalog No.: MA5-37888, Thermo Scientific USA) was diluted at a ratio of 1:400 in the antibody dilution. Other mediums and buffers were prepared as working solutions according to the requirements of the platform, with the exception of the primary HOXA9 antibody.

### Interpretation of results

The localization of the HOXA9 protein was primarily observed in the cytoplasm, with a lesser presence in the nucleus. Positive HOXA9 staining was characterized by the presence of brownish-yellow granules in the cytoplasm and/or nucleus of cancer cells. Two qualified pathologists conducted the examination of immunohistochemical staining results using a double-blind method. In cases of disagreement, a third pathologist re-evaluated the sections to determine a consensus score. The scoring system for HOXA9 immunostaining was defined as follows according to the method published in the literatures before(Yuan et al. 2018): absence of staining or positive staining in less than 5% of cells was denoted as “−”; positive staining in 5–25% of cells was denoted as “ + ”; positive staining in over 25% but less than 50% of cells was denoted as “++”; and positive staining in over 50% of cells was denoted as “+++”. Tissue sections with scores “++” and “+++” were classified as high HOXA9 expression, while those with “−” or “+” scores were classified as low HOXA9 expression.

### qRT-PCR assay

A total of 100 paraffin-embedded CRC tissues and 10 matched adjacent normal tissues, with available follow-up data, were utilized for the analysis of miR-140-3p and HOXA9 mRNA expression. The extraction of miRNA and mRNA was carried out using the respective extraction kits. Subsequently, the RNA was reverse transcribed into cDNA using the MonScriptTM miRNA First Strand cDNA Synthesis Kit (Tailing Reaction) following the provided instructions. The reaction system was prepared using the tailing method with cDNA as a template. PCR amplification was performed using an ABI Step One Plus Fluorescent PCR instrument (Applied Biosystems, USA) as instructed by the MonAmpTM ChemoHS qPCRMix kit. The qRT-PCR program was set as follows: 95 °C for 10 min (pre-denaturation) → 95 °C for 10 s (denaturation) → 60 °C for 10 s (annealing) → 72 °C for 30 s (extension) for 40 cycles. GAPDH and U6 were separately used as reference genes for mRNA and miRNA measurements. The primer sequences are provided in Table 1. The experiments were conducted in triplicates, and the expression levels of miR-140-3p and HOXA9 mRNA in the tested samples were calculated based on the Ct values obtained using the 2^(-ΔΔCt) relative quantification method, respectively.

**Table 1:** The primer sequences employed in this study

qRT-PCR primer sequences	
Primer name	Primer sequence (5′–3′)
HOXA9 forward primer	CCCTGACTGACTATGCTTGTGGTTC
HOXA9 reverse primer	CTTGTCTCCGCCGCTCTCATTC
GAPDH forward primer	AGATCCCTCCAAAATCAAGTGG
GAPDH reverse primer	GGCAGAGATGATGACCCTTTT
miR-140-3p forward prime	TACCACAGGGTAGAACCACGG
miR-140-3p reverse primer	GCAGGGTCCGAGGTATTC
U6 Forward prime	CTCGCTTCGGCAGCACA
U6 Reverse primer	AACGCTTCACGAATTTGCGT

### Follow-up visit

The follow-up period commenced on the day of surgery, and the endpoint for the follow-up visit was either the date of the patient’s death or June 30, 2022. Follow-up data of 193 cases were available for analysis. To assess the overall survival differences, a Kaplan–Meier overall survival curve was constructed.

### Statistical analysis

For data analysis, the SPSS 26.0 software was utilized. The qualitative variables were presented as the number of cases, and group comparisons were performed using the *χ*^2^ test. Quantitative variables were expressed as mean ± standard deviation, and group comparisons were conducted using the Student’s *t* test. The correlation between miR-140-3p and HOXA9 expression was evaluated using the Spearman’s rank correlation coefficient. The overall survival difference was analyzed using the log-rank test. Univariate and multivariable prognostic analysis was conducted using the COX proportional hazards regression model. A *p* value of less than 0.05 was considered statistically significant, indicating a significant difference between groups or associations between variables.

## Results

### miR-140-3p is downregulated in colorectal carcinoma

**Fig. 1:**
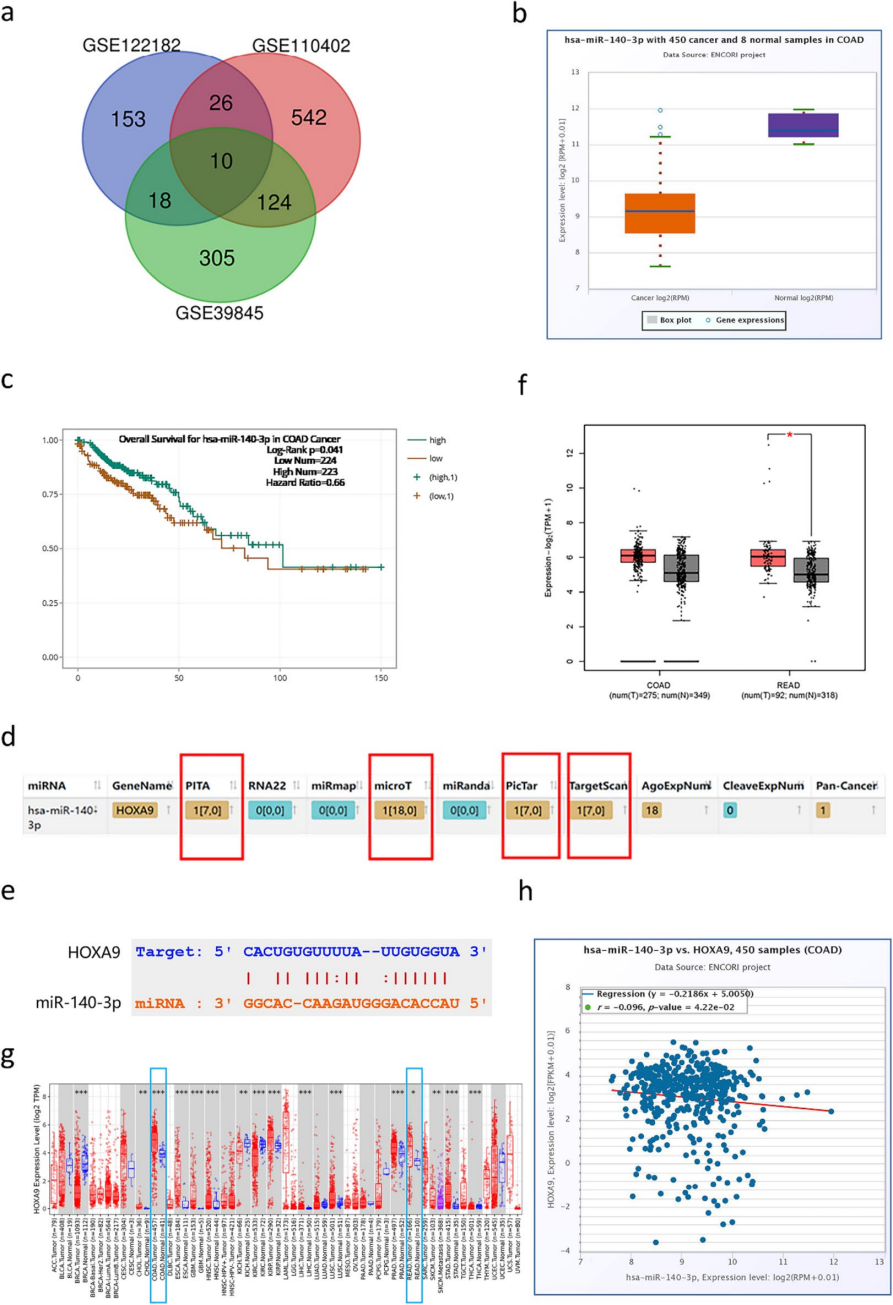
Bioinformatic analysis was conducted to investigate the expression of miR-140-3p and HOXA9 in colorectal cancer (CRC) and their correlation. **a** Differentially expressed miRNAs were identified by analyzing three datasets (GSE122182, GSE110402, and GSE39845) from the GEO database. Differentially expressed miRNAs were identified using the following criteria: |LogFc| > 1 and *p* < 0.01. Fc = expression of colon cancer tissue/adjacent normal tissue. **b** miR-140-3p expression was evaluated in colon cancer tissue samples (*n* = 450) and compared to normal colon tissue samples (*n* = 8). The data used for this analysis was obtained from ENCORI. **c** Overall survival analysis was performed on a cohort of 447 colon cancer patients using the ENCORI database. The Kaplan–Meier analysis revealed a significant decrease in cumulative survival among patients with low miR-140-3p expression (represented by the brown line) compared to those with high expression (represented by the green line). The statistical significance was determined using the log-rank test (*p* = 0.041). **d** Four miRNA target gene databases (PITA, microT, PicTar, and TargetScan) predicted HOXA9 as a target gene of miR-140-3p. The prediction data were generated via ENCORI. **e** The binding sites of miR-140-3p with HOXA9 were investigated. **f** HOXA9 mRNA expression was analyzed in tumor and normal tissue samples from patients with colonic and rectal adenocarcinoma. The dataset included 275 colon adenocarcinoma (COAD) tumor samples, 92 rectal adenocarcinoma (READ) tumor samples, 349 COAD normal tissue samples, and 318 READ normal tissue samples. The analysis was conducted using GEPIA2. **g** The expression profile of HOXA9 mRNA was examined in a pan-cancer context, specially focusing on its downregulation in tumor sections of COAD and READ. The analysis included 457 COAD tumor samples, 166 READ tumor samples, 41 COAD normal tissue samples, and 10 READ normal tissue samples. The data was obtained through TIMER. **h** In colon cancer, a negative correlation between miR-140-3p and HOXA9 expression was observed in a sample size of 450, with statistical significance (*p* < 0.05). This correlation data was generated through ENCORI

Three miRNA expression datasets (GSE122182, GSE110402, and GSE39845) were retrieved from the Gene Expression Omnibus (GEO) database. Differentially expressed miRNAs were identified using the following criteria: |LogFc| > 1 and *p* < 0.01. The overlapping miRNAs that showed differential expression in all three datasets were determined using the VENN drawer online tool (Fig. 1a). 10 miRNAs were found to be differentially expressed, namely miR-497, miR-139-5p, miR-140-3p, miR-30a-star, miR-29b-2-star, miR-30a, miR-143-star, miR-139-3p, miR-195, and miR-133a. While several of these miRNAs have been extensively studied in colorectal cancer (CRC) (Pidikova et al. 2020), the clinical application and regulation networks of miR-140-3p which is one of the most distinct miRNA and has reported having important biological functions in cellular level remain underexplored. Therefore, we focused on miR-140-3p for further investigation. Consistently, analysis of the ENCORI database confirmed that miR-140-3p is significantly downregulated in colon cancer tissues compared to paired normal tissues (Fig. 1b).

To investigate the clinical significance of dysregulated miR-140-3p expression, we performed survival curve analysis using the ENCORI database. The analysis revealed that patients with lower miR-140-3p expression, whose expression levels below the average, exhibited a significantly poorer prognosis (Fig. 1c). These findings suggest that miR-140-3p likely plays a crucial role in colorectal cancer and may have clinical relevance as a potential prognostic indicator for this disease.

### HOXA9 is a potential novel target of miR-140-3p

To elucidate the functional role of miR-140-3p, we employed the comprehensive ENCORI database to identify potential downstream target genes. Among the candidates, HOXA9 emerged as a highly scored and novel target which did not report before (Fig. 1d). Since miRNAs typically exert their functions by binding to target gene mRNA sequences to degrade mRNA or inhibit translation, we performed a sequence comparison and identified a significant binding region between miR-140-3p and HOXA9 mRNA (Fig. 1e), indicating that HOXA9 may be a potential binding target of miR-140-3p.

Additionally, we investigated the expression profiles of HOXA9 using the GEPIA2 database (Fig. 1f) and the TIMER database (Fig. 1g). Interestingly, contrary to miR-140-3p, HOXA9 exhibited upregulation in colon adenocarcinoma (COAD) and rectum adenocarcinoma (READ) tumor tissues. To further validate the relationship between miR-140-3p and HOXA9, we analyzed their correlation, and the ENCORI data revealed a negative correlation in colon cancer tissues (Fig. 1h).

In summary, our analysis demonstrates that miR-140-3p is downregulated, while HOXA9 is upregulated in tumor tissues compared to normal tissues, indicating an unfavorable prognosis. Importantly, miR-140-3p has the potential to bind to the mRNA sequence of HOXA9. These findings strongly suggest that HOXA9 represents a novel target of miR-140-3p, and miR-140-3p may regulate the development of colorectal cancer by targeting HOXA9.

### miR140-3p and HOXA9 axis is correlated with lymph node metastasis

**Fig. 2:**
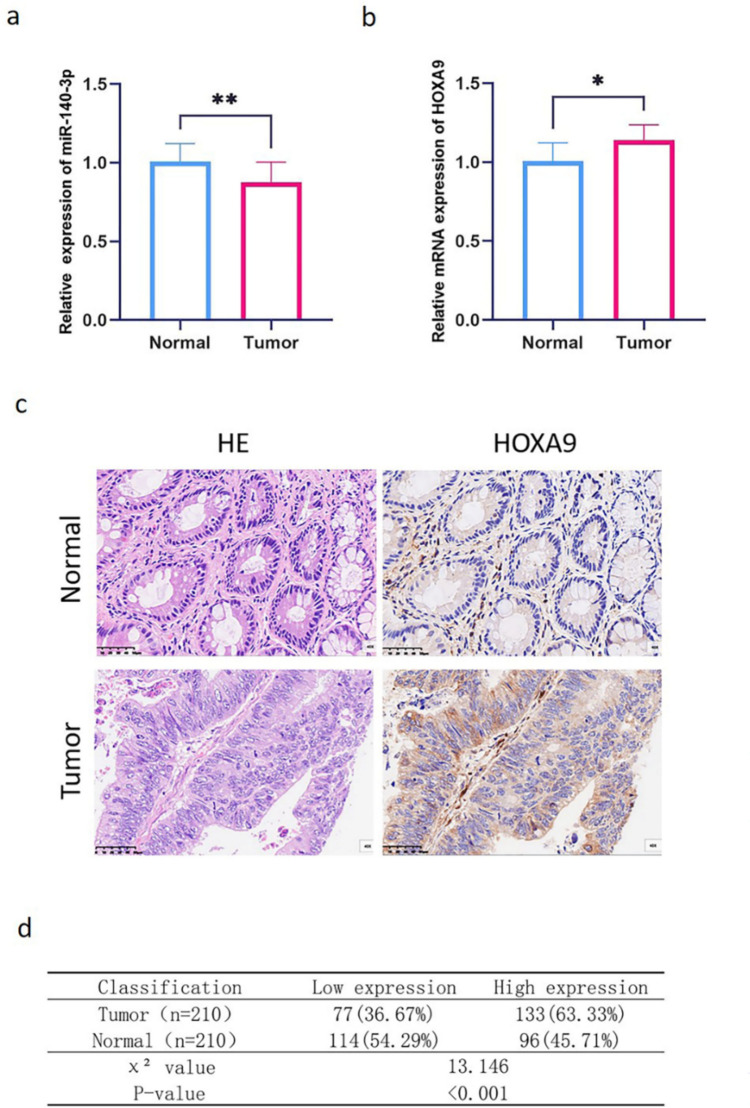
Expression analysis of miR-140-3p and HOXA9 in clinical samples. **a**, **b** The **a** illustrates the expression levels of miR-140-3p, while the **b** demonstrates the mRNA expression of HOXA9 in tumor and normal tissues obtained from clinical samples. **c** Representative images of hematoxylin and eosin (HE) staining and immunohistochemical staining of HOXA9 are provided to showcase the histopathological features and intensity and distribution of HOXA9 of both tumor and matched normal tissue samples captured at a magnification of ×40. **d** The statistical analysis presents quantitative data derived from the immunohistochemical staining of HOXA9. The results highlight a significant increase in the ratio of highly expressed HOXA9 within the tumor area

To validate the findings from bioinformatic analysis above, we conducted experimental assays using residual paraffin-embedded tissue samples in our institute. Quantitative reverse transcription-polymerase chain reaction (qRT-PCR) was performed to measure the expression levels of miR-140-3p and HOXA9. The results demonstrated that miR-140-3p expression was significantly downregulated in carcinoma tissues (0.87 ± 0.13, *n* = 100) compared to adjacent normal tissues (*n* = 10) (Fig. 2a). In contrast, HOXA9 mRNA expression was elevated in tumor tissues compared to matched normal tissues (1.14 ± 0.1, both *n* = 10) (Fig. 2b).

Immunohistochemical staining was performed to examine the protein expression of HOXA9 in colorectal cancer and paired normal tissues. The results revealed that HOXA9 protein was predominantly localized in the cytoplasm, with a smaller fraction detected in the nucleus (Fig. 2c). The high expression rate of HOXA9 protein was observed in colorectal cancer tissues compared to adjacent normal tissues (63.33%, 133 cases vs 45.71%, 96 cases) (Fig. 2d). In summary, our analysis of clinical samples confirmed the downregulation of miR-140-3p and the upregulation of HOXA9 in colorectal tumor tissues.

Furthermore, we investigated the relationship between miR-140-3p or HOXA9 expression and the clinicopathological characteristics of colorectal cancer patients. Based on the mean value of miR-140-3p expression, 100 randomly selected colorectal cancer patients were divided into the high expression group (36 cases) and the low expression group (64 cases). Similarly, based on immunohistochemical results for HOXA9, 210 colorectal cancer patients were divided into the low HOXA9 expression group (77 cases) and the high expression group (133 cases). The relationship between miR-140-3p and HOXA9 expression in colorectal cancer tissues and the clinicopathological characteristics of patients was analyzed. The results (Table 2) indicated that low miR-140-3p expression was associated with lymph node metastasis (*p* < 0.05), and high HOXA9 expression was associated with lymph node metastasis and lympho-vascular invasion (*p* < 0.05). However, miR-140-3p and HOXA9 expression levels showed no significant association with age, gender, differentiation, TNM stage, tumor diameter, and tumor type, tumor localization, KRAS mutation, p53 mutation and MSI status (*p* > 0.05). Ki67 expression correlated to some extent with miR-140-3p and HOXA9 expression, although the associations were not statistically significant (*p* = 0.078 and *p* = 0.091, respectively). In summary, the dysregulation of the miR-140-3p and HOXA9 axis in colorectal cancer is closely correlated with lymph node metastasis.

**Table 2:** Associations between miR-140-3p and HOXA9 expression levels and clinicopa-thological characteristics in patients diagnosed with colorectal cancer

Clinicopathological parameters	miR-140-3p				HOXA9			
	High expression	Low expression	*χ*^2^	*p*	High expression	Low expression	*χ*^2^	*p*
	(*n* = 36)	(*n* = 64)			(*n* = 133)	(*n* = 77)		
Gender			0.425	0.514			0.756	0.385
Male	21	33			66	43		
Female	15	31			67	34		
Age (years)			1.089	0.4			0.03	0.863
≤ 55	13	30			36	20		
> 55	23	34			97	57		
The degree of differentiation			0.168	0.682			1.397	0.237
High differentiation	15	24			63	30		
Low to medium differentiation	21	40			70	47		
Lymph node metastases			7.436	0.006			4.114	0.043
Yes	9	34			78	34		
No	27	30			55	43		
TNM staging			1.29	0.256			2.738	0.098
I–II	26	39			69	49		
III–IV	10	25			64	28		
Tumor size (cm)			0.967	0.325			1.904	0.168
≤ 4	20	29			77	37		
> 4	16	35			56	40		
Tumor site			1.582	0.453			1.857	0.113
Left hemicolon	2	6			14	2		
Right hemicolon	12	27			32	20		
Sigmoid/rectum	22	31			87	55		
Approximate type			4.278	0.118			1.894	0.388
Raised type	3	3			14	4		
Ulcerative type	31	61			118	72		
Infiltrative type	2	0			1	1		
Lympho-vascular invasion			2.042	0.153			4.187	0.041
Yes	7	21			55	21		
No	29	43			78	56		
KRAS mutation			0.174	0.677			0.974	0.324
Yes	12	24			45	21		
No	24	40			88	56		
P53 mutation			0.005	0.943			0.962	0.367
Yes	25	44			95	50		
No	11	20			38	27		
Ki67 proliferative index			3.241	0.078			2.952	0.091
≤ 60%	8	9			20	16		
> 60%	28	55			113	61		
Microsatellite status			1.316	0.251			2.573	0.109
MSS	33	62			128	70		
MSI	3	2			5	7		

### HOXA9 is an independent prognosis factors for colorectal cancer patients

**Fig. 3:**
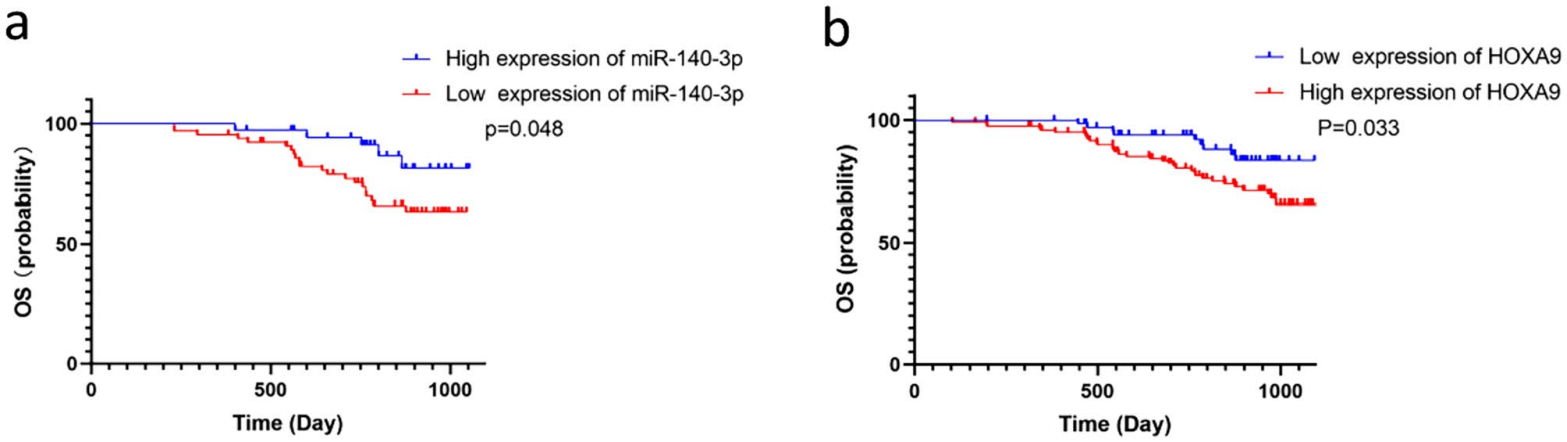
Prognostic analysis of miR-140-3p and HOXA9 expression in colorectal cancer patients. **a** Among our cohort of colorectal cancer patients, the miR-140-3p low expression group (*n* = 64) exhibited a significantly poorer prognosis in comparison to the miR-140-3p high expression group (*n* = 36) (*p* = 0.048). **b** Similarly, within our colorectal cancer patient cohort, the HOXA9 high expression group (*n* = 122) demonstrated a significantly unfavorable prognosis compared to the HOXA9 low expression group (*n* = 71) (*p* = 0.033)

The Kaplan–Meier survival curves were generated by our cohort to assess the effect of miR-140-3p (Fig. 3a) and HOXA9 (Fig. 3b) expression on the prognosis of colorectal cancer patients. The results revealed that the 3-year overall survival rate was 86.1% (31 case/36 case) in the miR-140-3p high expression group, while the miR-140-3p low expression group exhibited significantly poorer outcomes (3 years OS: 67.2%, 43 case /64 case) (*p* < 0.05). Similarly, for HOXA9, the 3-year overall survival rate was 73% (89 cases/122 cases) in the high expression group and 87.3% (62 cases/77 cases) in the low expression group, indicating a worse prognosis in the high HOXA9 expression group compared to the low expression group (*p* < 0.05).

Cox univariate regression analysis (Table 3) demonstrated that, in addition to the known prognostic factors such as differentiation, TNM stage, and lymph node metastasis, miR-140-3p and HOXA9 expression levels also significantly influenced the prognosis of colorectal cancer patients. Further, Cox multivariate regression analysis, after adjusting for potential confounding factors (Table 3), identified TNM stage and HOXA9 as independent risk factors for the prognosis of colorectal cancer patients, emphasizing their clinical significance.

**Table 3:** Univariate and Multifactorial Cox regression analysis investigating the prognostic factors influencing the outcomes of patients with colorectal cancer

Index	Univariate analysis			Multivariate analysis		
	HR	95% CI	*p *value	HR	95% CI	*p *value
Gender	1.175	0.277–1.192	0.137			
Age	1.002	0.966–1.033	0.95			
The degree of differentiation	1.282	1.195–1.881	0.022	1.036	0.971–1.204	0.096
Approximate type (ref = raised type)			0.553			
Ulcerative type	1.298	0.051–2.853	0.548			
Infiltrative type	1.363	0.049–2.711	0.324			
Tumor size	1.411	0.435–1.783	0.724			
Lympho-vascular invasion	1.289	0.684–2.072	0.26			
TNM staging	2.503	1.523–2.928	0.001	2.213	1.263–2.576	0.017
Lymph node metastases	1.462	1.026–2.547	0.035	1.197	0.323–1.501	0.356
HOXA9	2.191	1.367–2.412	0.04	1.345	1.062–1.973	0.042
miR-140-3p	0.813	0.318–0.925	0.048	0.974	0.286–1.717	0.436

In conclusion, low miR-140-3p expression and high HOXA9 expression were associated with poor prognosis in colorectal cancer patients. Notably, HOXA9 emerged as an independent risk factor for the prognosis of these patients, highlighting its potential as a prognostic biomarker in colorectal cancer management.

## Discussion

Colorectal cancer (CRC) is a significant malignant tumor with a high mortality rate due to recurrence and metastasis, despite advancements in treatment modalities such as surgery, radiotherapy, chemotherapy, and immunotherapy (Dekker et al. 2019; Kennedy et al. 2011). Biomarkers are crucial tools for CRC diagnosis, classification, and prognostic prediction. MiRNAs have emerged as important players in tumor pathogenesis, diagnosis, and treatment, prompting us to screen for miRNAs that can serve as markers and targets for CRC diagnosis and treatment. In this study, we identified miR-140-3p as an important miRNA in CRC development and uncovered HOXA9 as its novel target.

miR-140-3p has been to some extend investigated in other cancer types, often functioning as a tumor suppressor. For instance, low miR-140-3p expression in gastric cancer patients has been associated with poor prognosis and unfavorable clinicopathological features. Overexpression of miR-140-3p inhibits gastric cancer cell migration, invasion, and proliferation (Wang et al. 2021). Similarly, in bladder cancer, miR-140-3p hinders cancer cell proliferation and invasion by directly targeting FOXQ1 (Wang et al. 2020b). However, miR-140-3p can also act as a tumor promoter, as demonstrated in ovarian cancer, where it inhibits NK cell recruitment and diminishes cytotoxic effects, thereby promoting ovarian cancer cell growth through the mediation of MAPK1 (Wang et al. 2020a).

HOXA9, along with other AbdBC-type HOXA genes, plays a crucial role as a transcription factor in hematopoiesis and leukemogenesis (Abramovich et al. 2005; de Bock et al. 2018). Elevated HOXA9 expression has also been observed in breast cancer. In ovarian cancer, HOXA9 expression induces normal peritoneal fibroblasts to adopt a cancer-associated fibroblast (CAF) phenotype, stimulating the growth of ovarian cancer and endothelial cells (Ko et al. 2012). Additionally, HOXA9 has been implicated in chemotherapy resistance to 5-fluorouracil in CRC (Ju et al. 2017). However, studies have shown that HOXA9 hypermethylation promotes hepatocellular carcinoma development (Kuo et al. 2014), suggesting that HOXA9 may have different roles in different cancer types.

In our study, bioinformatic analysis revealed low expression of miR-140-3p and high expression of HOXA9 in colorectal cancer tissues. Furthermore, miR-140-3p was found to regulate HOXA9 by binding to its mRNA, a finding that was confirmed through examination of clinical samples. Our limitation lies in not directly verifying their interaction, which would have provided favorable evidence of their connection. Clinical data demonstrated that low miR-140-3p expression and high HOXA9 expression were associated with lymph node metastasis and poor prognosis in CRC patients. Notably, HOXA9 emerged as an independent risk factor for colorectal cancer patients. It is intriguing that in our multivariate analysis, miR-140-3p did not emerge as an independent risk factor. This absence of significance could be attributed to the robust interaction observed between miR-140-3p and lymph node metastasis, as illustrated in Table 2. In multivariate Cox analysis, the assessment of a variable’s significance takes place while considering other variables concurrently. It is plausible that the significance of miR-140-3p and lymph node metastasis diminished when accounting for their interactions or dependencies with other covariates. Consequently, these factors did not demonstrate statistical significance in the multivariate Cox analysis.

Indeed, miR-140-3p and HOXA9 exhibit substantial functional overlap and share common targets. Both miR-140-3p and HOXA9 play crucial roles in cell stemness, proliferation, apoptosis, and epithelial-mesenchymal transition (EMT). For instance, in breast cancer, the downregulation of miRNA-140 promotes proliferation and inhibits apoptosis by influencing SOX2 expression (Zhang et al. 2012). Intriguingly, HOXA9 synergistically regulates SOX2 and BCL2 to suppress apoptosis and mediate leukemogenesis (Miyamoto et al. 2021). In colorectal cancer (CRC), BCL2 is recognized as a direct target of miR140-3p (Liu et al. 2021). It has been shown that miR-140-3p regulates the PI3K/AKT, JAK/STAT, mTOR, TGFβ/Smad, and Wnt pathways in tumors (Taheri et al. 2023), while HOXA9 influences the expression levels of PI3K/AKT, JAK/STAT, mTOR, NF-kB, HIF1a, and the Wnt pathway (Tang et al. 2022). Therefore, miR-140-3p and HOXA9 may engage in crosstalk at various cellular levels.

Our data showed miR-140-3p and HOXA9 predict the poor prognosis and indicated its expression related to clinical lymph node metastasis. Previous studies have pointed that the Wnt pathway is a shared signaling of both miR-140-3p and HOXA9, playing a crucial role in the EMT process (Xu et al. 2021, Gonzalez and Medici 2014). Bioinformatic analysis in CRC has revealed a positive relationship between HOXA9 and β-catenin (gene: CTNNB1), while miR140-3p exhibits the opposite trend (Supplementary Fig. 1). Consequently, we hypothesize that the miR-140-3p-HOXA9 axis may influence lymph node metastasis and contribute to poor outcomes through the Wnt/β-catenin-mediated EMT process, necessitating further exploration in future studies.

## Supplementary Information

Below is the link to the electronic supplementary material.

Supplementary file1 (PDF 150 KB)

## Data Availability

The raw data used to support the findings of this study are available from the corresponding author upon reasonable request.

## References

[CR1] Abramovich C, Pineault N, Ohta H, KeithHumphries R (2005) Hox genes: from leukemia to hematopoietic stem cell expansion. Ann N Y Acad Sci 1044:109–116 15958703 10.1196/annals.1349.014

[CR2] Chen X, Zeng K, Xu M, Liu X, Hu X, Xu T, He B, Pan Y, Sun H, Wang S (2019a) P53-induced miR-1249 inhibits tumor growth, metastasis, and angiogenesis by targeting VEGFA and HMGA2. Cell Death Dis 10:131. 10.1038/s41419-018-1188-3 30755600 10.1038/s41419-018-1188-3 PMC6372610

[CR3] Chen Y, Deng X, Chen W, Shi P, Lian M, Wang H, Wang K, Qian D, Xiao D, Long H (2019b) Silencing of microRNA-708 promotes cell growth and epithelial-to-mesenchymal transition by activating the SPHK2/AKT/beta-catenin pathway in glioma. Cell Death Dis 10:448. 10.1038/s41419-019-1671-5 31171769 10.1038/s41419-019-1671-5 PMC6554356

[CR4] Ciesla M, Skrzypek K, Kozakowska M, Loboda A, Jozkowicz A, Dulak J (2011) MicroRNAs as biomarkers of disease onset. Anal Bioanal Chem 401:2051–2061. 10.1007/s00216-011-5001-8 21544542 10.1007/s00216-011-5001-8

[CR5] de Bock CE, Demeyer S, Degryse S, Verbeke D, Sweron B, Gielen O, Vandepoel R, Vicente C, Vanden Bempt M, Dagklis A, Geerdens E, Bornschein S, Gijsbers R, Soulier J, Meijerink JP, Heinaniemi M, Teppo S, Bouvy-Liivrand M, Lohi O, Radaelli E, Cools J (2018) HOXA9 cooperates with activated JAK/STAT signaling to drive leukemia development. Cancer Discov 8:616–631. 10.1158/2159-8290.CD-17-0583 29496663 10.1158/2159-8290.CD-17-0583

[CR6] Dekker E, Tanis PJ, Vleugels JLA, Kasi PM, Wallace MB (2019) Colorectal cancer. Lancet 394:1467–1480. 10.1016/S0140-6736(19)32319-0 31631858 10.1016/S0140-6736(19)32319-0

[CR7] Dong W, Yao C, Teng X, Chai J, Yang X, Li B (2016) MiR-140-3p suppressed cell growth and invasion by downregulating the expression of ATP8A1 in non-small cell lung cancer. Tumour Biol 37:2973–2985. 10.1007/s13277-015-3452-9 26415732 10.1007/s13277-015-3452-9

[CR8] Gonzalez DM, Medici D (2014) Signaling mechanisms of the epithelial-mesenchymal transition. Sci Signal 7:re8. 10.1126/scisignal.2005189 25249658 10.1126/scisignal.2005189 PMC4372086

[CR9] Ji D, Chen Z, Li M, Zhan T, Yao Y, Zhang Z, Xi J, Yan L, Gu J (2014) MicroRNA-181a promotes tumor growth and liver metastasis in colorectal cancer by targeting the tumor suppressor WIF-1. Mol Cancer 13:86. 10.1186/1476-4598-13-86 24755295 10.1186/1476-4598-13-86 PMC4021214

[CR10] Jiang W, Li T, Wang J, Jiao R, Shi X, Huang X, Ji G (2019) miR-140-3p suppresses cell growth and induces apoptosis in colorectal cancer by targeting PD-L1. Onco Targets Ther 12:10275–10285. 10.2147/OTT.S226465 31819512 10.2147/OTT.S226465 PMC6885546

[CR11] Ju T, Jin H, Ying R, Xie Q, Zhou C, Gao D (2017) Overexpression of NAC1 confers drug resistance via HOXA9 in colorectal carcinoma cells. Mol Med Rep 16:3194–3200. 10.3892/mmr.2017.6986 28713930 10.3892/mmr.2017.6986 PMC5547960

[CR12] Kennedy RD, Bylesjo M, Kerr P, Davison T, Black JM, Kay EW, Holt RJ, Proutski V, Ahdesmaki M, Farztdinov V, Goffard N, Hey P, McDyer F, Mulligan K, Mussen J, O’Brien E, Oliver G, Walker SM, Mulligan JM, Wilson C, Winter A, O’Donoghue D, Mulcahy H, O’Sullivan J, Sheahan K, Hyland J, Dhir R, Bathe OF, Winqvist O, Manne U, Shanmugam C, Ramaswamy S, Leon EJ, Smith WI Jr, McDermott U, Wilson RH, Longley D, Marshall J, Cummins R, Sargent DJ, Johnston PG, Harkin DP (2011) Development and independent validation of a prognostic assay for stage II colon cancer using formalin-fixed paraffin-embedded tissue. J Clin Oncol 29:4620–4626. 10.1200/JCO.2011.35.4498 22067406 10.1200/JCO.2011.35.4498

[CR13] Ko SY, Barengo N, Ladanyi A, Lee JS, Marini F, Lengyel E, Naora H (2012) HOXA9 promotes ovarian cancer growth by stimulating cancer-associated fibroblasts. J Clin Invest 122:3603–3617. 10.1172/JCI62229 22945634 10.1172/JCI62229 PMC3461910

[CR14] Kuo CC, Lin CY, Shih YL, Hsieh CB, Lin PY, Guan SB, Hsieh MS, Lai HC, Chen CJ, Lin YW (2014) Frequent methylation of HOXA9 gene in tumor tissues and plasma samples from human hepatocellular carcinomas. Clin Chem Lab Med 52:1235–1245. 10.1515/cclm-2013-0780 24681432 10.1515/cclm-2013-0780

[CR15] Li DP, Li ZY, Sang W, Cheng H, Pan XY, Xu KL (2013) HOXA9 gene expression in acute myeloid leukemia. Cell Biochem Biophys 67:935–938. 10.1007/s12013-013-9586-8 23575938 10.1007/s12013-013-9586-8

[CR16] Li J, Zhao J, Wang H, Li X, Liu A, Qin Q, Li B (2018) MicroRNA-140-3p enhances the sensitivity of hepatocellular carcinoma cells to sorafenib by targeting pregnenolone X receptor. Onco Targets Ther 11:5885–5894. 10.2147/OTT.S179509 30271172 10.2147/OTT.S179509 PMC6149869

[CR17] Liu D, Chen C, Cui M, Zhang H (2021) miR-140-3p inhibits colorectal cancer progression and its liver metastasis by targeting BCL9 and BCL2. Cancer Med 10:3358–3372. 10.1002/cam4.3840 33838016 10.1002/cam4.3840 PMC8124101

[CR18] Miyamoto R, Kanai A, Okuda H, Komata Y, Takahashi S, Matsui H, Inaba T, Yokoyama A (2021) HOXA9 promotes MYC-mediated leukemogenesis by maintaining gene expression for multiple anti-apoptotic pathways. Elife. 10.7554/eLife.64148 34310280 10.7554/eLife.64148 PMC8313233

[CR19] Paczek S, Lukaszewicz-Zajac M, Mroczko B (2022) Granzymes—their role in colorectal cancer. Int J Mol Sci. 10.3390/ijms23095277 35563668 10.3390/ijms23095277 PMC9104098

[CR20] Pidikova P, Reis R, Herichova I (2020) miRNA clusters with down-regulated expression in human colorectal cancer and their regulation. Int J Mol Sci. 10.3390/ijms21134633 32610706 10.3390/ijms21134633 PMC7369991

[CR21] Sung H, Ferlay J, Siegel RL, Laversanne M, Soerjomataram I, Jemal A, Bray F (2021) Global Cancer Statistics 2020: GLOBOCAN estimates of incidence and mortality worldwide for 36 cancers in 185 countries. CA Cancer J Clin 71:209–249. 10.3322/caac.21660 33538338 10.3322/caac.21660

[CR22] Taheri F, Ebrahimi SO, Heidari R, Pour SN, Reiisi S (2023) Mechanism and function of miR-140 in human cancers: a review and in silico study. Pathol Res Pract 241:154265. 10.1016/j.prp.2022.154265 36509008 10.1016/j.prp.2022.154265

[CR23] Talarmain L, Clarke MA, Shorthouse D, Cabrera-Cosme L, Kent DG, Fisher J, Hall BA (2022) HOXA9 has the hallmarks of a biological switch with implications in blood cancers. Nat Commun 13:5829. 10.1038/s41467-022-33189-w 36192425 10.1038/s41467-022-33189-w PMC9530117

[CR24] Tang L, Peng L, Tan C, Liu H, Chen P, Wang H (2022) Role of HOXA9 in solid tumors: mechanistic insights and therapeutic potential. Cancer Cell Int 22:349. 10.1186/s12935-022-02767-9 36376832 10.1186/s12935-022-02767-9 PMC9664671

[CR25] Wang Y, Chen J, Wang X, Wang K (2020b) miR-140-3p inhibits bladder cancer cell proliferation and invasion by targeting FOXQ1. Aging (albany NY) 12:20366–20379. 10.18632/aging.103828 33098639 10.18632/aging.103828 PMC7655201

[CR26] Wang Z, Chen K, Li D, Chen M, Li A, Wang J (2021) miR-140-3p is involved in the occurrence and metastasis of gastric cancer by regulating the stability of FAM83B. Cancer Cell Int 21:537. 10.1186/s12935-021-02245-8 34656115 10.1186/s12935-021-02245-8 PMC8520196

[CR27] Wang J, Zhu M, Zhou X, Wang T, Xi Y, Jing Z, Xi W (2020a) MiR-140–3p inhibits natural killer cytotoxicity to human ovarian cancer via targeting MAPK1. J Biosci 45 32385217

[CR28] Watanabe Y, Saito M, Saito K, Matsumoto Y, Kanke Y, Onozawa H, Hayase S, Sakamoto W, Ishigame T, Momma T, Ohki S, Takenoshita S (2018) Upregulated HOXA9 expression is associated with lymph node metastasis in colorectal cancer. Oncol Lett 15:2756–2762. 10.3892/ol.2017.7650 29435001 10.3892/ol.2017.7650 PMC5778893

[CR29] Xu Q, Zhang Q, Dong M, Yu Y (2021) MicroRNA-638 inhibits the progression of breast cancer through targeting HOXA9 and suppressing Wnt/beta-cadherin pathway. World J Surg Oncol 19:247. 10.1186/s12957-021-02363-7 34416888 10.1186/s12957-021-02363-7 PMC8379838

[CR30] Yuan Y, Sun S, Jiao N, Shu Y, Zhang Y (2018) Upregulation of HOXA10 protein expression predicts poor prognosis for colorectal cancer. Genet Test Mol Biomark 22:390–397. 10.1089/gtmb.2017.0240 10.1089/gtmb.2017.0240 29870276

[CR31] Zhang Y, Eades G, Yao Y, Li Q, Zhou Q (2012) Estrogen receptor α signaling regulates breast tumor-initiating cells by down-regulating miR-140 which targets the transcription factor SOX2. J Biol Chem 287:41514–41522. 10.1074/jbc.M112.404871 23060440 10.1074/jbc.M112.404871 PMC3510847

[CR32] Zhou Y, Wang B, Wang Y, Chen G, Lian Q, Wang H (2019) miR-140-3p inhibits breast cancer proliferation and migration by directly regulating the expression of tripartite motif 28. Oncol Lett 17:3835–3841. 10.3892/ol.2019.10038 30881504 10.3892/ol.2019.10038 PMC6403497

