# Supplementary material for: Exploring the expression and clinical significance of the miR-140-3p-HOXA9 axis in colorectal cancer
Source: J Cancer Res Clin Oncol. 2024 Jan 29;150(2):47. doi: 10.1007/s00432-023-05592-3 (PMC10824855; doi:10.1007/s00432-023-05592-3)

Supplementary Fig 1: In colorectal cancer (CRC), miR-140-3p exhibited a negative correlation with CTNNB1 mRNA expression, while HOXA9 showed a positive correlation with CTNNB1 mRNA expression.

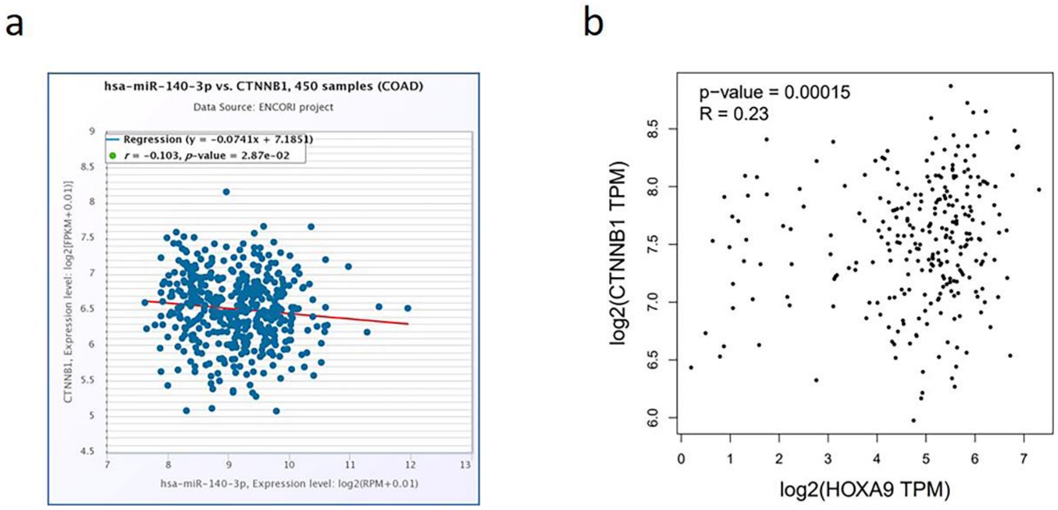

Supplement: Supplementary file 1 — Supplementary file1 (PDF 150 KB) [file 432_2023_5592_MOESM1_ESM.pdf]
